# Red Blood Cells’ Thermodynamic Behavior in Neurodegenerative Pathologies and Aging

**DOI:** 10.3390/biom11101500

**Published:** 2021-10-12

**Authors:** Svetla Todinova, Sashka Krumova, Desislava Bogdanova, Avgustina Danailova, Elena Zlatareva, Nikolay Kalaydzhiev, Ariana Langari, Ivan Milanov, Stefka G. Taneva

**Affiliations:** 1 Institute of Biophysics and Biomedical Engineering, Bulgarian Academy of Sciences, Acad. G. Bonchev, 1113 Sofia, Bulgaria; todinova@abv.bg (S.T.); sakrumo@gmail.com (S.K.); avgustina_danailova@abv.bg (A.D.); arianaiilias@abv.bg (A.L.); 2 Department of Neurology, University Multiprofile Hospital for Active Treatment in Neurology and Psychiatry Sv. Naum, 1113 Sofia, Bulgaria; dessislava_bogdanova@abv.bg (D.B.); el.zlatareva@gmail.com (E.Z.); kalaydzhiev_nikolay@abv.bg (N.K.); milanovivan@yahoo.com (I.M.)

**Keywords:** neurodegenerative diseases, red blood cells, hemoglobin, Band 3 protein, differential scanning calorimetry, thermal transitions, transition temperature, excess heat capacity

## Abstract

The main trend of current research in neurodegenerative diseases (NDDs) is directed towards the discovery of novel biomarkers for disease diagnostics and progression. The pathological features of NDDs suggest that diagnostic markers can be found in peripheral fluids and cells. Herein, we investigated the thermodynamic behavior of the peripheral red blood cells (RBCs) derived from patients diagnosed with three common NDDs—Parkinson’s disease (PD), Alzheimer’s disease (AD), and amyotrophic lateral sclerosis (ALS) and compared it with that of healthy individuals, evaluating both fresh and aged RBCs. We established that NDDs can be differentiated from the normal healthy state on the basis of the variation in the thermodynamic parameters of the unfolding of major RBCs proteins—the cytoplasmic hemoglobin (Hb) and the membrane Band 3 (B3) protein. A common feature of NDDs is the higher thermal stability of both Hb and B3 proteins along the RBCs aging, while the calorimetric enthalpy can distinguish PD from ALS and AD. Our data provide insights into the RBCs thermodynamic behavior in two complex and tightly related phenomena—neurodegenerative pathologies and aging, and it suggests that the determined thermodynamic parameters are fingerprints of the altered conformation of Hb and B3 protein and modified RBCs’ aging in the studied NDDs.

## 1. Introduction

Neurodegenerative diseases (NDDs) are an extremely important medical and social problem in today’s society. The prevalence of NDDs, particularly the most common Parkinson’s (PD) and Alzheimer’s (AD) diseases as well as amyotrophic lateral sclerosis (ALS), has continuously increased in recent years, partly due to an increase in life expectancy but also due to the lack of criteria for early diagnosis and still mainly symptomatic treatment [1,2,3]. In most cases, the causes of NDDs are idiopathic; a role in the etiology of these disorders is being played by the presence of environmental toxins, genetic predisposition, and the processes of oxidative stress and inflammation associated with the aging of the body.

There are still no reliable biomarkers identifying the complex pathways contributing to the NDDs and especially for their early diagnosis. The challenge of the current NDDs research is the discovery of new non-invasive biomarkers to identify individuals at risk or to monitor the disease progress and the patient’s response to therapy [4,5,6].

The pathological features of these diseases suggest that markers can also be found in peripheral blood cells and fluids, which stimulate the research efforts in this direction [7,8,9,10,11,12,13,14]. Data from the meta-analysis of all such studies indicate that no circulating markers in the blood have been detected so far in concentrations allowing differentiation, for example, of AD patients from control individuals [15,16].

This pilot study aimed to identify novel non-invasive biomarkers for three NDDs—PD, ALS, and AD based on the thermodynamic behavior of the most abundant peripheral blood cells—red blood cells (RBCs).

Human RBCs are unique cells, deprived not only of a nucleus but also of all subcellular organelles, including mitochondria [17]. In structural terms, the cell is maintained by a system of membrane cytoskeleton, consisting of a two-dimensional submembranous meshwork of spectrin tetramers connected by actin junctional complexes and anchored to the lipid bilayer by “binding complexes” [18,19,20]. The unbound parts of the membrane cytoskeleton are flexible and render elasticity, flexibility, and a dynamic change in the morphology of RBCs during their passage into the bloodstream [17,21]. The binding complexes contain the B3 protein, a transmembrane glycoprotein which plays a major role in the RBC membrane integrity, in cell metabolism, and in the exchange of oxygen with hemoglobin (Hb), the major cytoplasmic constituent of RBCs, and tissues [22,23,24]. A change in a single component of the membrane cytoskeleton can lead to a modification of the whole structure, which would impair the cells’ function and the mechanism of oxygen transport. All tissues depend on the RBCs function, especially neurons, which use 20% of the total consumed oxygen.

In this work, we applied a biophysical approach based on differential scanning calorimetry (DSC) to explore for the first time the thermodynamic stability of the major RBCs functional proteins (spectrin; Band 2.1, 4.1, and 4.2 proteins; Band 3 (B3) protein; and hemoglobin (Hb)) in the selected NDDs pathologies and to compare it with healthy individuals. We expected that the thermodynamic behavior of major RBC proteins and, accordingly, the parameters: denaturation temperature (T_m_), excess heat capacity (c_P_^ex^) of their unfolding, and the enthalpy (ΔH_cal_) of the calorimetric profile would be affected in the investigated NDDs.

Since NDDs are age-related diseases determining the thermodynamic characteristics of RBCs, aging is also of special interest [25,26]. Therefore, we followed the biophysical characteristics of the NDDs RBCs during their aging pathway in the search of variations that distinguish them from those of healthy cells that we have recently characterized [27].

We identified thermodynamic features of unfolding of the major RBCs proteins, Hb and B3 protein, that distinguish the neurodegenerative disorders from normal healthy state.

## 2. Materials and Methods

### 2.1. Patients and Baseline Characteristics

Patients with NDDs were diagnosed at the university multiprofile hospital for active treatment in neurology and psychiatry “St. Naum” (UMHATNP), Sofia. The Ethics Committee for research investigations at UMHATNP approved this study (Consent number 05/15.03.2018) in agreement with the principles of the Declaration of Helsinki of 1975, revised in 2013 for research involving human subjects. All patients provided written consent. Baseline characteristics, age, gender, creatinine level, hemoglobin, and hematocrit (Htc), were gathered.

### 2.2. RBCs Preparation

Blood samples were derived by venipuncture at the UMHATNP from 20 patients with NDDs and 9 healthy donor volunteers. RBCs were separated by centrifugation at 3000 rpm for 15 min at 4 °C and washed three times with PBS buffer (10 mM sodium phosphate, pH 7.2, 140 mM NaCl, and 1 mM ethylenediaminetetraacetic acid (EDTA)). The obtained RBCs were characterized by DSC. For the aging experiments, the cells were stored at 4 °C at 30% Htc and washed with PBS buffer directly before use.

### 2.3. Differential Scanning Calorimetry

The calorimetric profiles (thermograms) of RBCs from patients with NDDs and healthy subjects were recorded using a DASM 4 microcalorimeter in the range of 30–100 °C with a scanning rate of 1 °C/min. RBCs were suspended in PBS to a final Hb concentration of 8 mg/mL, which was determined spectrophotometrically. The following thermodynamic parameters were determined: temperature, T_m_, and excess heat capacity, c_P_^ex^, of the transitions in the calorimetric profiles corresponding to spectrin, band 2.1, 4.1, and 4.2 proteins; B3 protein and Hb; and the calorimetric enthalpy (the integrated area under the endothermic transitions of the heat capacity profile), ΔH_cal_, of the thermograms. All data were analyzed by an Origin software routine.

### 2.4. Statistical Analysis

DSC results are presented as mean ± standard error (SE). The statistical significance of the difference between the thermodynamic parameters (T_m_ and ΔH_cal_ values) of the three NDDs and healthy sets was determined by performing a non-parametric statistical test (Mann–Whitney U test function embedded in OriginPro 2018 software). *p* values < 0.05 were considered significant. Statistical analysis was not performed for AD patients because of the low number of cases.

## 3. Results

### 3.1. Patient Characteristics

Twenty patients with NDDs were selected on clinical criteria. Nine of the patients fulfilled the 2015 MDS-PD clinical criteria and were diagnosed with PD [28]. Eight patients were selected based on the El Escorial criteria and diagnosed with ALS—five of them were with clinically definite and three with clinically probable and laboratory supported forms [29]. Three of the investigated patients were with probable AD [30]. PD patients with comorbid dementia and AD patients with depression were excluded. The eight healthy donors were not smokers and were selected not to have neurodegenerative, hereditary or another disease. The baseline characteristics, age, gender, creatinine concentration, RBCs indices (total Hb, hematocrit (Hct) determined according standard clinical procedures), are summarized in Table 1.

The Hct level in RBCs from the studied healthy controls, PD, and ALS patients was in the range of the normal Hct level (38.3–48.6% for M and 35.5–44.9% for F), while in AD cells it was slightly lower than the lower cut-off value (Table 1). The serum creatinine level (considered as potential marker for NDDs) was within the cut-off values (60 to 110 μmol/L for M and 45 to 90 μmol/L for F) for most of patients and had a higher value (109.5 and 125.6 μmol/L) than the upper cut-off only for two female PD patients (average values are given in Table 1). The creatinine level was not measured for the healthy donors.

### 3.2. Thermodynamic Behavior of Proteins in Fresh RBCs

**Figure 1:**
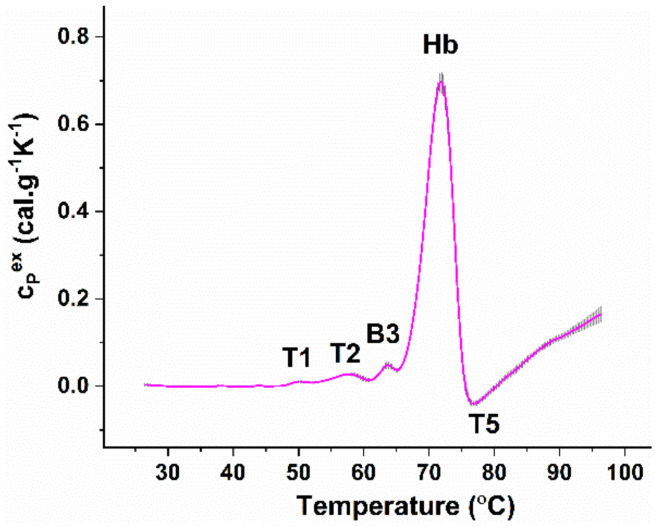
Average set of DSC profiles of freshly isolated RBCs from nine healthy donors heated with a 1°C/min scanning rate; mean profile (magenta solid line) and standard error (grey shadow). Hb concentration was set to 8 mg/mL. For clarity, the successive thermal transitions are denoted (see text for details).

The temperature-induced unfolding of the components of freshly isolated (i.e., measured at the day of obtaining the blood samples) healthy RBCs is composed of four endothermic thermal transitions followed by an exothermic one, presented in Figure 1. The endothermic transitions were previously assigned to the following cell components: spectrin (the first transition T1 localized at ca. 50 °C); bands 2.1, 4.1, and 4.2 proteins (transition T2 at 57°); the membrane-spanning domain of B3 glycoprotein (transition B3 at ca. 63 °C); and Hb (the major transition, denoted Hb, at 72 °C) [27,31,32,33,34]. The exothermic transition (T5 at ca. 78 °C) was found to be due to Hb post-denaturation aggregation.

**Figure 2:**
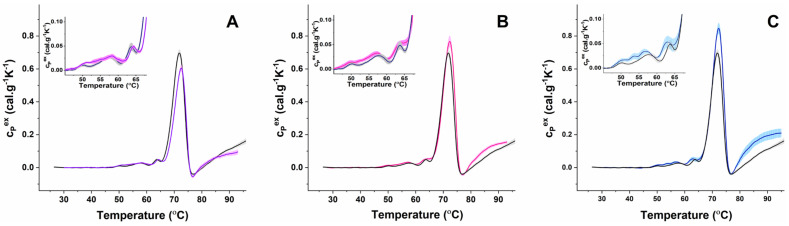
Mean DSC profiles of freshly isolated RBCs from nine PD ((**A**) blue line and magenta shadow, SE), eight ALS ((**B**) magenta line and pink shadow, SE) and three AD ((**C**) blue line, blue shadow, SE) patients plotted together with that from nine healthy volunteers (black line, gray shadow, SE). Data were obtained for samples with Hb concentration of 8 mg/mL at a heating rate of 1 °C/min. Insets represent zooms of the temperature range where the cytoskeletal and membrane proteins melt.

The same number of thermal transitions were resolved in the calorimetric profiles of fresh RBCs from PD-, ALS-, and AD-diagnosed patients (Figure 2). The obtained calorimetric profiles were compared with the healthy ones in terms of T_m_ and c_P_^ex^ values of the resolved calorimetric transitions.

**Figure 3:**
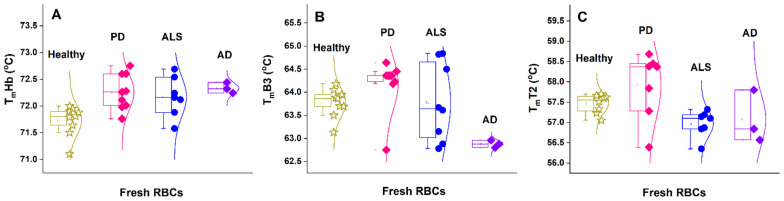
Transition temperature of the unfolding of Hb (**A**), B3 protein (**B**), and transition T2 (assigned to Band 2.1, 4.1, and 4.2 proteins) (**C**) of freshly isolated RBCs from healthy (asterisks) subjects compared to cells from PD (squares), ALS (circles), and AD (diamond) patients.

The results showed that in all three types of diseased cells the mean T_m_ values of the Hb transition were slightly higher than those obtained for the healthy controls (Figure 3A and Table 2). However, the amplitude of the Hb transition (c_P_^ex^Hb) depended strongly on the disease and had a lower value for PD, while it had a higher value for ALS and AD cells compared to healthy ones (Figure 2 and Table 2).

The low temperature range (below 65 °C), where proteins of the cytoskeleton and the plasma membrane unfold, was altered in fresh NDDs cells (Figure 2 insets). For PD cases, T1 and T2 transitions were broader and not as well defined as in the control set. The mean T_m_ of T2 transition ascribed to Band 2.1, 4.1, and 4.2 proteins was downshifted in ALS and AD cells and slightly higher for PD cases, compared to the healthy one (Figure 3C and Table 2). It is to be noted that T_m_ values of T2 transition for PD cases dispersed more than those of ALS and AD cases (Figure 3C). For AD cases, a transition at 54 °C was resolved with a so-far-unidentified nature (Figure 2C).

The shape of the B3 protein transition was preserved for PD cases but was broader for ALS and AD cases (Figure 2, insets). The mean T_m_ of this transition was slightly upshifted in the PD and downshifted in AD (Figure 3B), but it was not shifted in ALS compared to that in healthy RBCs profiles (Table 2).

The calorimetric enthalpy of the thermograms, ΔH_cal_, had a lower value for PD, while they had a higher value for ALS and AD RBCs relative to the healthy one (Table 2); thus, they followed the same trend as that of the c_P_^ex^ of Hb and B3 transitions. This strongly suggests that different energy is required for proteins’ unfolding in PD, ALS, and AD RBCs compared to healthy ones.

### 3.3. Aging Effect on the Thermodynamic Behavior of Diseased vs. Healthy RBCs

In line with our previous work (Dinarelli et al. [27]), the T_m_ and c_P_^ex^ of Hb and B3 protein thermal transitions in healthy cells were strongly changed during aging, and both proteins became less stable against the thermal treatment (series of the DCS profiles for healthy RBCs along aging are presented in Supplementary Figure S1).

**Figure 4:**
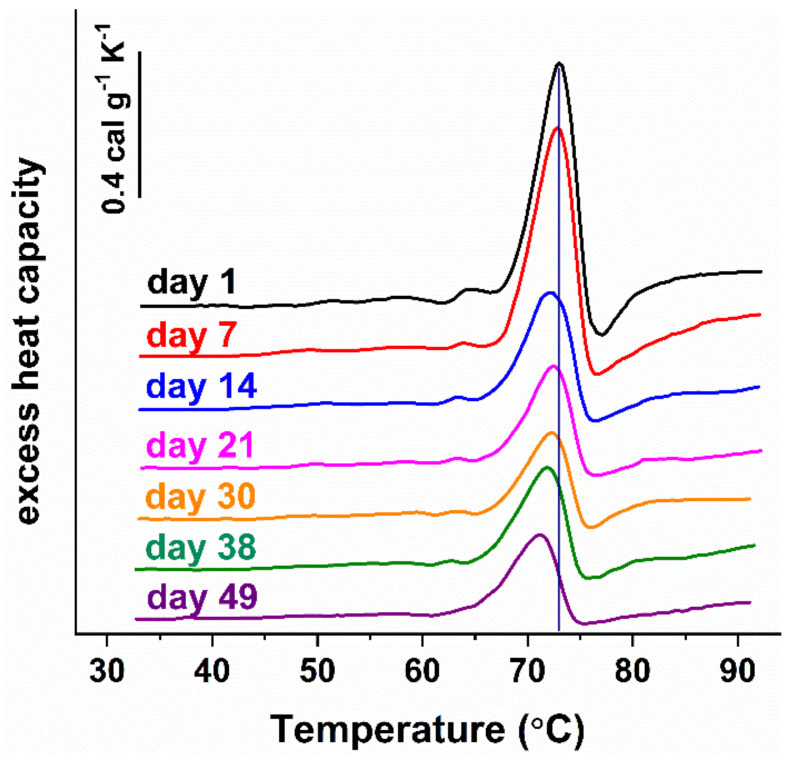
Representative series of DSC profiles of RBCs derived from PD patient and recorded for a period of 49 days. RBCs with Hb concentration of 8 mg/mL were heated with 1°C/min scanning rate. The transition temperature of Hb in freshly prepared RBCs (day 1) is marked by a vertical line.

**Figure 5:**
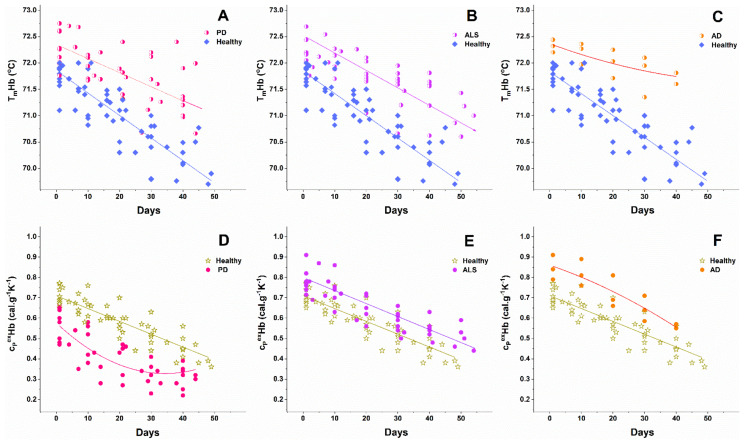
Change in the transition temperature (**A**–**C**) and excess heat capacity (**D**–**F**) of Hb transition along the aging of RBCs from healthy individuals and patients with PD (**A**,**D**), ALS (**B**,**E**), and AD (**C**,**F**). Symbols are shown in the corresponding panels.

**Figure 6:**
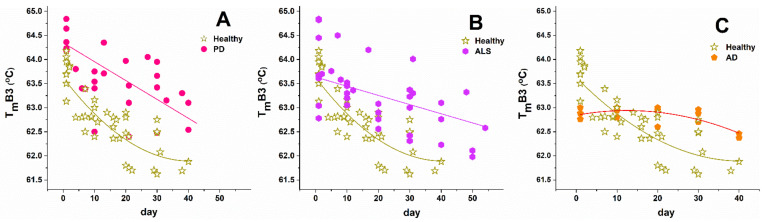
Change in the transition temperature of B3 unfolding along the aging of RBCs. Data for healthy individuals are compared with those for PD (**A**), ALS (**B**), and AD (**C**) cases. Symbols are shown in the corresponding panels.

The thermodynamic behavior of NDDs cells was also affected by the cells’ aging (representative calorimetric profiles recorded along the aging process of RBCs from one PD case are shown in Figure 4). The series of calorimetric profiles recorded along NDD cells’ aging are presented in Supplementary Figures S2–S4 for the studied PD, ALS, and AD cases, respectively. Like healthy RBCs, the NDDs (PD, ALS, and AD) cells became less stable against thermal treatment (Figure 5 and Figure 6) and the excess heat capacity c_P_^ex^ of Hb (Figure 5) and B3 protein unfolding (data not shown) in all studied cells that decreased along the aging process.

The T_m_ of Hb transition decreased linearly along the aging of healthy and NDDs cells (Figure 5), while that of B3 protein decreased exponentially for healthy and linearly for the diseased cells (Figure 6). With the onset of the ageing process (after the 1st day) the T_m_ values of both Hb and B3 transitions were higher for PD, ALS, and AD patients than those determined for healthy controls (Figure 5 and Figure 6).

Along the aging, the amplitude of the Hb transition remained higher for ALS and AD cells than for healthy ones, with the effect being more pronounced for AD cells. The Hb transition amplitude in PD cells, however, was lower than that of healthy cells along the whole studied period (Figure 5D–F).

**Figure 7:**
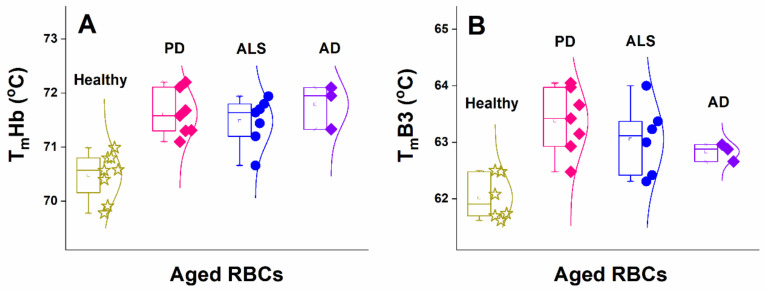
Temperature of Hb (**A**) and B3 protein (**B**) unfolding of aged (29-day- to 32-day-aged) RBCs from healthy (asterisks) subjects compared with cells from PD (squares), ALS (circles), and AD (diamond) patients.

Comparing in more detail the T_m_s of Hb and B3 protein, determined for aged RBCs, it became clear that in aged diseased cells both proteins were thermally more stable than in healthy cells, and the mean T_m_ of both proteins was higher for aged NDD cells compared to healthy cells (Figure 7 and Table 3).

Moreover, the difference between the T_m_s of aged and fresh cells for both Hb and B3 protein was more pronounced for healthy controls than for NDDs cases. The T_m_ difference between fresh and ca. 30-day-aged healthy cells was 1.2 °C and 1.8 °C for Hb and B3 protein, respectively, and 2 °C for both Hb and B3 protein between fresh and 40-day-aged healthy cells (Table 3). This difference in T_m_s was ca. 0.6 °C and 1 °C for Hb, and ca. 0.8 °C and 1.6 °C for B3 protein of 30-day- and 40-day-aged PD cells, respectively (Table 3). The ALS RBCs were somewhat more stable against thermal treatment compared to PD cells; the Tm difference was estimated to be ca. 0.8–1 °C for Hb and B3 protein after 30 and 40 days of cells’ aging (Table 3 and Figure 7). Among the three studied NDDs and the healthy controls, both Hb and B3 protein were most stable in AD RBCs, the Tm difference of Hb between the fresh and aged ALS cells being ca. 0.5–0.6 °C, while the B3 protein stability was not changed up to day 40 of the aging process (Table 3).

The transitions T1 and T2 were difficult to evaluate because of their too low amplitude in aged RBCs.

Like for fresh PD, the calorimetric enthalpy of aged PD RBCs had a lower mean value and, for aged ALS and AD, had higher values than that of healthy cells (Table 3). Statistical analysis showed that the differences of the PD and ALS thermodynamic parameters (T_m_ of B3 protein and Hb and ΔH_cal_) relative to those of healthy cells were significant (*p* < 0.05), and analysis was not performed for the AD set because of the low number of cases.

## 4. Discussion

Over the last decade, DSC has been applied in investigation of complex biofluids such as blood plasma/sera [35,36,37,38,39,40,41,42,43,44,45,46,47,48,49,50], cerebrospinal fluid [51,52], cancer cells and nuclei [53], and brain tissue [54]. Farkas et al. [55,56] have demonstrated that DSC can well be used to extract information on drug effects on blood plasma and RBCs [55,56] as well as on F and G actin in polyneuropathy [57]. A recent study of Michnik et al. [58] evaluated the usefulness of DSC in differentiating the severity of PD based on a considerable difference in the thermodynamic parameters of protein denaturation in comparison to healthy sera.

To the best of our knowledge, no data have been published so far specifically regarding the thermodynamic properties of RBCs in pathological conditions, such as PD, AD, and ALS. As mentioned above, we have already successfully explored DSC for the analysis of healthy RBCs’ thermodynamic behavior along the cells’ aging and revealed age-dependent changes in the main thermodynamic parameters of Hb and B3 protein [27]. In this pilot study, we expanded our scope and reported on a direct comparison between the thermodynamic behavior of fresh and aged RBCs derived from patients with PD, ALS, and AD and those from healthy individuals. In particular, we revealed NDDs-induced effects on the thermal stability of Hb and B3 proteins that are reflected in the RBCs calorimetric profiles.

Although the number of studied AD cases is very low, it helped us deduce whether neurodegenerative disorders with a different nature might exhibit similar effects on the thermal stability of RBC. Indeed, we clearly demonstrate for the first time that a common feature of the three studied neurodegenerative disorders is the higher thermal stability of Hb in NDDs cells than in healthy ones—this effect was slightly expressed in fresh cells but became clearly noticeable with cells’ aging. The T_m_Hb difference was of ca. 2 °C for 40-day-aged NDD cells relative to control ones, while for fresh cells this increment was less than 1 °C. It should be noted that although small, in thermodynamic terms, these differences are significant. Besides, the amplitude of the Hb transition was changed in the order c_P_Hb^PD^ < c_P_Hb^healthy^ < c_P_Hb^ALS^ < c_P_Hb^AD^ for fresh RBCs and along their aging (Figure 5D–F).

A similar trend was observed for T_m_ of B3 protein—while, in fresh cells, it was close to the control, and along the aging it became progressively higher for the NDDs’ cases relative to the control values. Importantly, both Hb and B3 protein are most stable in AD cells and most unstable in healthy cells.

Changes in the calorimetric features of spectrin in NDDs and control cells were also observed in fresh RBCs but could not be characterized precisely in the course of cells’ aging due to the low amplitude of this transition and respective biases in its resolution.

The enthalpy of the calorimetric curves of fresh RBCs from the three studied NDDs differed from that of the healthy cells (Table 2) and had higher values for ALS and AD, while it had a lower value for PD cases than for the healthy one. This tendency was also seen for the enthalpy of aged NDDs cells relative to healthy ones. This suggests that different energy is required to disrupt the interactions that stabilize the structure of Hb and membrane proteins and to unfold them in either fresh or aged healthy and NDDs cells.

The altered stability of RBCs’ proteins in the three studied NDDs suggests significant modification in their conformation, which was reflected in the recorded calorimetric curves. Conceivably, the shifts we observed in the unfolding temperatures of those major RBCs proteins might result from their significantly different binding state considering the well-known presence of low levels of misfolded peptides in RBCs as well as in blood plasma [59,60], which might directly interact with RBC and plasma proteins [61]. In particular, NDDs are characterized by abnormal accumulation and misfolding of specific proteins, mainly β-amyloid peptide (40 to 42 amino acids long, Aβ1-40 and Aβ1-42), τ-protein, and α-synuclein (α-syn), both in the brain and in peripheral tissues. A mixed model of proteinopathies in the brain of patients affected by NDD has been identified [61], showing that in addition to homoaggregates, α -syn, τ, and Aβ interact with each other or with other “pathological proteins” and form toxic heteroaggregates in the brains and peripheral blood cells of patients [4,12,59,62,63]. Therefore, the presence of amyloids and heterocomplexes circulating in the bloodstream that can bind to RBC proteins is a possible reason for the altered binding state/conformation and thermodynamics of unfolding of Hb and B3 protein.

Importantly, the similar effect of RBCs’ aging on Hb and B3 protein stability for PD, ALS, and AD, i.e., linear Tm vs. aging time dependences, points to a common aging pathway of both proteins for the three studied disorders that is in line with the suggested possibility of a unified mechanism of development of neurodegenerative disorders and their relation to the aging process in the recent review of Scheiblich et al. [25]. However, we established a different aging pattern, which was exponential, for the Tm of B3 protein in healthy cells. This is indicative of different aging pathways of the membrane B3 protein in NDDs and healthy cells and thus age-induced changes in the cell membrane structure and the cytoskeleton.

It is known that B3 protein interacts with Hb and with other membrane proteins, thus regulating the RBCs’ flexibility and stability [17,23] and building an interaction network responsible for signals’ transduction. This interaction has been shown to be particularly important for triggering the removal of aged RBCs from the blood circulation under hypoxic conditions [64]. Shakalai et al. [65] characterized the Hb binding to RBCs membrane and provided evidence for two types (low and high affinity) of binding sites. In fresh RBCs, little or no interaction was observed between Hb and the outer cell surface [65]. However, proteomic analyses suggest that aging is associated with increased binding of B3 protein and (modified) Hb, modified B3 conformation, disruption of the cytoskeleton, and formation of vesicles [66]. We can hypothesize that the modified network of interaction in the NDDs cells might cause modified stability and flexibility of the RBCs proteins and might even lead to delayed removal of the aged NDDs cells (characterized with an impaired function) from the circulation.

## 5. Conclusions

The calorimetric approach was successfully applied to differentiate healthy and NDDs RBCs. The data clearly showed that the thermodynamic parameters of unfolding of the major RBCs proteins, Hb and B3, in NDDs were distinct from those in healthy cells, the effect being more pronounced for aged than for fresh cells. On the other hand, PD can be distinguished from ALS on the basis of the calorimetric enthalpy in both fresh and aged cells. Therefore, these parameters reflect altered conformation and/or binding states of RBC proteins and might serve as promising biomarkers for NDDs. Further investigations in a larger cohort of patients are needed to validate the DSC approach as a new strategy to identify biomarkers for NDDs.

## Figures and Tables

**Table 1:** Clinical characteristics of the PD, ALS, and AD patients; mean values ± SD.

Clinical Characteristics	Healthy	PD	ALS	AD
Number of patients	9	9	8	3
Mean age (in years) ± S.D.	58.8 ± 10.2	68.2 ± 11.2	62.6 ± 12.8	75 ± 7
(Range, years)	(42–76)	(47–86)	(42–78)	(70–83)
Gender, M/F	3/6	4/5	5/3	0/3
Creatinine (μmol/L)	-	90.5 ± 17.2	61.6 ± 12.0	55.1 ± 15
Hct (%)	41.3 ± 2.1	38.0 ± 4.0	39.3 ± 4.8	30.5 ± 7.8
Hb (g/L)	137.2 ± 6.6	134.4 ± 15.2	140.0 ± 20.2	107.6 ± 37.2

**Table 2:** Transition temperatures (T_m_ (°C)) and excess heat capacities (c_P_^ex^ (cal.g^−1^K^−1^)) of the resolved the endothermic transitions (mean value ± SE) in fresh RBCs from healthy individuals and PD, ALS, and AD patients.

T_m_T1	T_m_T2	T_m_B3	T_m_Hb	ΔH_cal_
(c_P_^ex^T1)	(c_P_^ex^T2)	(c_P_^ex^B3)	(c_P_^ex^Hb)	
**Healthy**				
50.0 ± 0.06	57.5 ± 0.08	63.7 ± 0.1	71.7 ± 0.09	3.78 ± 0.1
(0.011 ± 0.002)	(0.027 ± 0.002)	(0.047 ± 0.003)	(0.71 ± 0.01)	
**PD**				
51.2 ± 0.05	57.9 ± 0.3	64.2 ± 0.2	72.3 ± 0.11	3.30 ± 0.14 *
(0.016 ± 0.002)	(0.030 ± 0.003)	(0.049 ± 0.002)	(0.58 ± 0.02)	
**ALS**				
49.7 ± 0.08	57.0 ± 0.12	63.8 ± 0.3	72.2 ± 0.14	4.36 ± 0.15 *
(0.014 ± 0.001)	(0.033 ± 0.003)	(0.054 ± 0.004)	(0.78 ± 0.02)	
**AD**				
50.05 ± 0.	57.0 ± 0.37	62.9 ± 0.04	72.3 ± 0.06	4.69 ± 0.3 *
(0.015 ± 0.004)	(0.035 ± 0.004)	(0.053 ± 0.01)	(0.85 ± 0.03)	

Asterisks denote a statistically significant difference of the enthalpy ΔHcal of NDD groups vs. healthy group (* *p* < 0.5).

**Table 3:** Temperature (Tm (°C)) of unfolding of B3 protein and Hb, and calorimetric enthalpy (ΔHcal (cal.g^−1^)) of the endothermic transition of aged RBCs (29-day- to 32-day-aged and 40-day-aged cells). Mean values ± SE.

T_m_B3		T_m_Hb		ΔH_cal_	
30 Day	40 Day	30 Day	40 Day	30 Day	40 Day
**Healthy**					
62.02 ± 0.1	61.78 ± 0.1	70.48 ± 0.2	69.64 ± 0.5	3.21 ± 0.2	2.72 ± 0.3
**PD**					
63.38 ± 0.2 ****	62.64 ± 0.2	71.61 ± 0.2 **	71.28 ± 0.1 ***	2.64 ± 0.3 ***	2.00 ± 0.1 *
**ALS**					
63.06 ± 0.2 ****	62.69 ± 0.2	71.48 ± 0.1 **	71.40 ± 0.2 ***	3.62 ± 0.1 ***	3.28 ± 0.4 ****
**AD**					
62.83 ± 0.1	62.42 ± 0.0	71.79 ± 0.2	71.70 ± 0.1	3.40 ± 0.4	3.45 ± 0.1

Asterisks denote statistically significant difference of the thermodynamic parameters (Tm and ΔHcal) of NDD groups vs. healthy group (* *p* ≤ 0.04–0.048; ** *p* ≤ 0.03–0.04; *** *p* ≤ 0.02–0.03; **** *p* < 0.002).

## Data Availability

All data are presented in the article.

## Supplementary Materials

The following are available online at https://www.mdpi.com/article/10.3390/biom11101500/s1. Sets of the series of DSC profiles of RBCs recorded along the cells aging are presented in Supplementary Figures: Figure S1. Healthy donors (denoted HC1–HC9); Figure S2. PD patients (denoted PD1–PD9); Figure S3. ALS patients (denoted ALS1–ALS8); Figure S4. AD patients (denoted AD1–AD3).
