# Supplementary material for: Red Blood Cells’ Thermodynamic Behavior in Neurodegenerative Pathologies and Aging
Source: Biomolecules. 2021 Oct 12;11(10):1500. doi: 10.3390/biom11101500 (PMC8534019; doi:10.3390/biom11101500)
Supplement: Supplementary file 1 [file biomolecules-11-01500-s001.zip › biomolecules-1385305-supplementary.pdf]

Supplementary Material

Red blood cells thermodynamic behavior in neurodegenerative pathologies and aging

Svetla Todinova[1], Sashka Krumova[1], Desislava Bogdanova[2], Avgustina Danailova[1], Elena Zlatareva[2], Nikolay Kalaydzhiev[2], Ariana Langari[1], Ivan Milanov[2], Stefka G. Taneva[1,*]

[1]Institute of Biophysics and Biomedical Engineering, Bulgarian Academy of Sciences, "Acad. G. Bonchev" Str. 21, 1113 Sofia, Bulgaria
[2]University multiprofile hospital for active treatment in neurology and psychiatry "Sv. Naum", Louben Roussev Str. 1, Sofia 1113, Bulgaria

Supplementary Figures

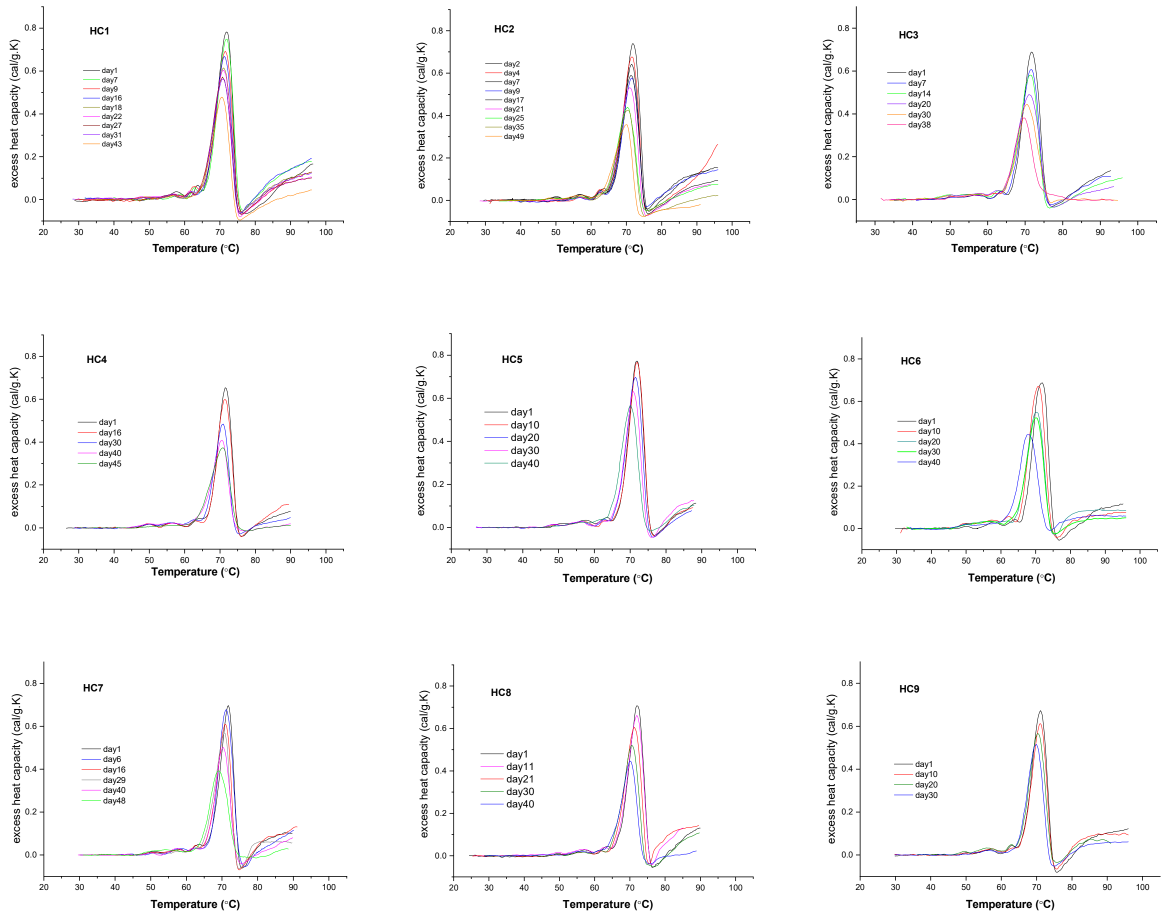

Supplementary Figure S1. Series of calorimetric curves of normal healthy RBCs measured along the cells aging (denoted HC1 – HC9), the days of the DSC measurements are indicated for each healthy donor in the corresponding panel.

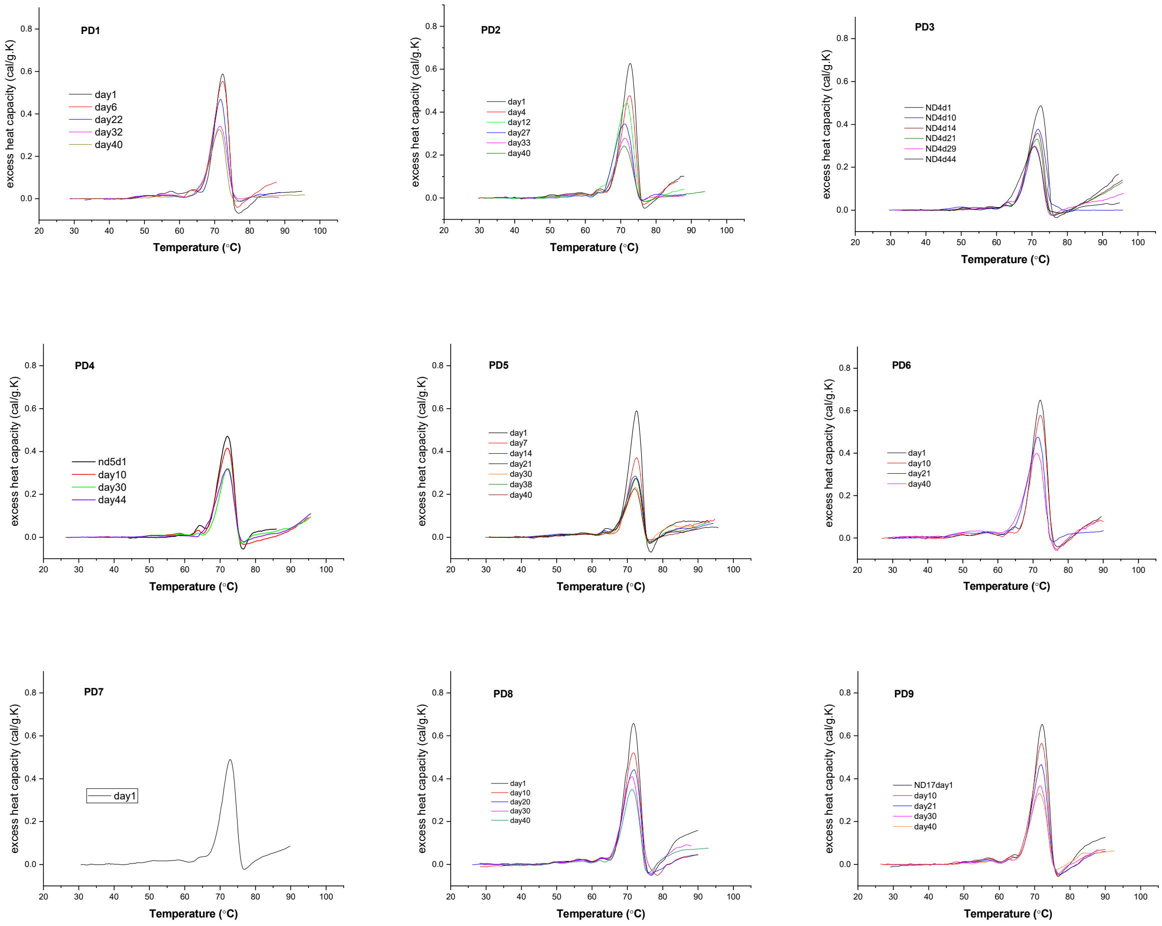

Supplementary Figure S2. Series of calorimetric curves of RBCs derived from the nine PD patients along the cells aging (denoted PD1 – PD9), for each studied PD case the days of the DSC measurements are indicated in the corresponding panel.

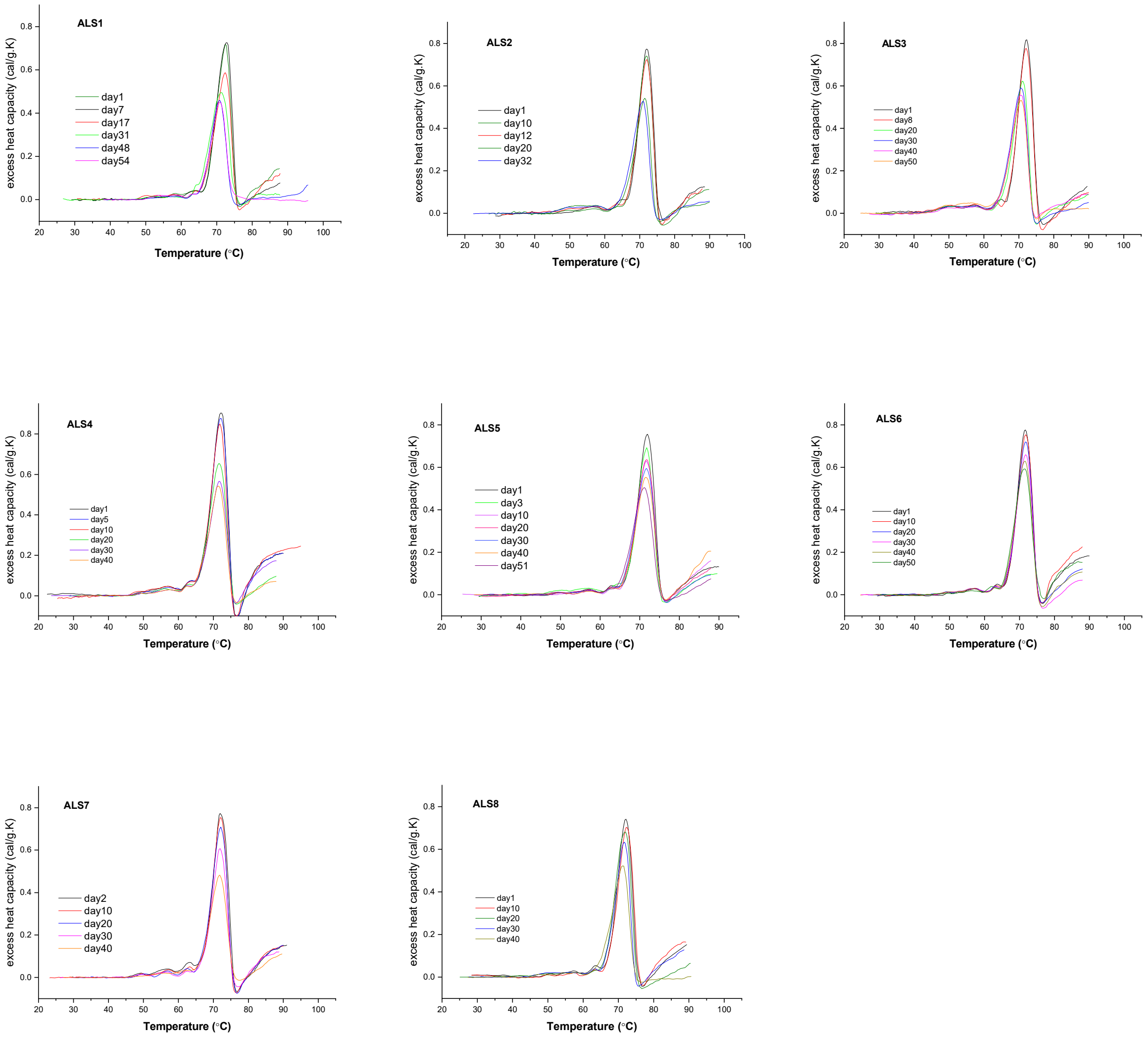

Supplementary Figure S3. Series of calorimetric curves of RBCs derived from the eight ALS patients during cells aging (denoted ALS1 – ALS8), the days of the DSC measurements are indicated for each studied ALS case in the corresponding panel.

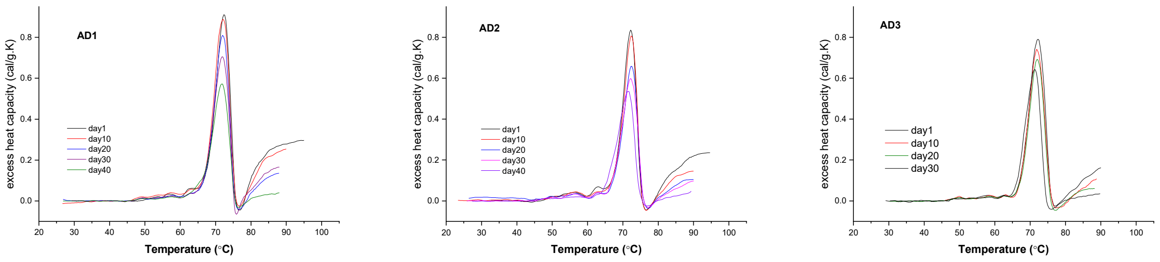

Supplementary Figure S4. Series of calorimetric curves of RBCs derived from three AD patients along the cells aging (denoted AD1 – AD3), the days of the DSC measurements are indicated for each studied AD case in the corresponding panel.
